# Improved characteristics of near-band-edge and deep-level emissions from ZnO nanorod arrays by atomic-layer-deposited Al_2_O_3 _and ZnO shell layers

**DOI:** 10.1186/1556-276X-6-556

**Published:** 2011-10-17

**Authors:** Wen-Cheng Sun, Yu-Cheng Yeh, Chung-Ting Ko, Hau He, Miin-Jang Chen

**Affiliations:** 1 Department of Materials Science and Engineering, National Taiwan University, Taipei 10617, Taiwan; 2 Graduate Institute of Photonics and Optoelectronics, National Taiwan University, Taipei 10617, Taiwan

## Abstract

We report on the characteristics of near-band-edge (NBE) emission and deep-level band from ZnO/Al_2_O_3 _and ZnO/ZnO core-shell nanorod arrays (NRAs). Vertically aligned ZnO NRAs were synthesized by an aqueous chemical method, and the Al_2_O_3 _and ZnO shell layers were prepared by the highly conformal atomic layer deposition technique. Photoluminescence measurements revealed that the deep-level band was suppressed and the NBE emission was significantly enhanced after the deposition of Al_2_O_3 _and ZnO shells, which are attributed to the decrease in oxygen interstitials at the surface and the reduction in surface band bending of ZnO core, respectively. The shift of deep-level emissions from the ZnO/ZnO core-shell NRAs was observed for the first time. Owing to the presence of the ZnO shell layer, the yellow band associated with the oxygen interstitials inside the ZnO core would be prevailed over by the green luminescence, which originates from the recombination of the electrons in the conduction band with the holes trapped by the oxygen vacancies in the ZnO shell.

PACS 68.65.Ac; 71.35.-y; 78.45.+h; 78.55.-m; 78.55.Et; 78.67.Hc; 81.16.Be; 85.60.Jb.

## Introduction

Because of large surface-to-volume ratio and spatial confinement of carriers, researches on one-dimensional (1D) nanostructures have attracted great interest [1-3], and remarkable progress has been achieved in various electronic, photonic, and sensing devices [3-7]. Novel synthetic approaches to the fabrication of high-quality semiconductor nanotubes have been reviewed by Yan et al. [8]. Zinc oxide (ZnO) has been regarded as one of the most promising materials for 1D nanostructures due to its distinguished characteristics such as direct and wide band gap (approximately 3.37 eV), large excitonic binding energy (up to 60 meV), and high piezoelectricity [9-11]. The synthesis of well-aligned ZnO nanorod arrays (NRAs) is crucially important for the practical applications such as field emitters [12], nanogenerators [13], solar cells [14], and nanolasers [15]. One of the popular techniques for fabricating ZnO NRAs is to use Au as catalyst on a lattice-matched substrate [16]. Since the optical properties of ZnO NRAs are strongly dependent on surface conditions [17-20] and natural defect states [21-24], a large variety of surface modifications on ZnO NRAs have been carried out by depositing a shell layer. For instance, the enhancement of photoluminescence (PL) has been observed in ZnO/Er_2_O_3 _and ZnO/MgZnO core-shell NRAs [25,26]. The enhanced surface-excitonic emission together with the suppression in deep-level emission has also been reported in ZnO/amorphous-Al_2_O_3 _core-shell nanowires [27]. Apart from the enhancement of light emission, strong photoconductivity [28], photocatalytic activity [29], and quantum confinement [30] have been observed in various 1D ZnO nanostructures.

In this paper, vertically aligned ZnO NRAs were synthesized using an aqueous chemical method, which is beneficial for low reaction temperature, low cost, catalyst-free synthesis, and large-scale production. The growth of ZnO NRAs was assisted by a ZnO seed layer prepared by atomic layer deposition (ALD). The self-limiting and layer-by-layer growth of ALD contribute to many advantages such as easy and accurate thickness control, conformal step coverage, high uniformity over a large area, low defect density, good reproducibility, and low deposition temperature. Therefore, highly conformal Al_2_O_3 _and ZnO shell layers could be deposited upon the surface of ZnO nanorods by ALD to form the ZnO/Al_2_O_3 _and ZnO/ZnO core-shell NRAs in this study. PL measurements were conducted to investigate the optical characteristics of ZnO/Al_2_O_3 _and ZnO/ZnO core-shell NRAs. The near-band-edge (NBE) emission was significantly enhanced, and the deep-level band was suppressed by the Al_2_O_3 _and ZnO shells due to the flat-band effect and the reduction in the surface defect density. In addition, the shift of deep-level emissions from the yellow band to the green band in ZnO/ZnO core-shell structure was reported. The mechanisms of flat-band effect and the shift of deep-level emissions were elucidated in detail.

## Experimental details

The ZnO NRAs were synthesized on (100) Si wafers by aqueous chemical growth. Before the synthesis, a 50-nm-thick ZnO seed layer was deposited on the wafer at a temperature of 180°C by ALD. Diethylzinc and H_2_O vapors were used as the precursors for zinc and oxygen, respectively. After the ALD deposition, the seed layer was treated by rapid thermal annealing at 950°C for 5 min in nitrogen atmosphere to improve its crystal quality. Afterwards, the ZnO NRAs were grown in 320 ml aqueous solution, containing 10 mM zinc nitrate hexahydrate and 5 ml ammonia solution, at 95°C for 2 h. More details of ZnO NRA synthesis have been described elsewhere [31,32]. Finally, Al_2_O_3 _and ZnO shell layers were prepared by the ALD on the as-grown ZnO NRAs to fabricate ZnO/Al_2_O_3 _and ZnO/ZnO core-shell NRAs. The precursors for Al_2_O_3 _deposition were trimethylaluminum and H_2_O vapors, and the deposition temperature was 180°C. The Al_2_O_3 _shell layers were 2, 5, and 10 nm in thickness. The ALD condition of ZnO shell layers was the same as that of the ZnO seed layer. The thicknesses of ZnO shell layers were 5, 10, and 15 nm, respectively. The details of ZnO and Al_2_O_3 _ALD parameters can be found in our previous studies [33-35].

The structural characterization of ZnO NRAs was examined by Germini LEO 1530 field emission scanning electron microscopy (SEM) (**Carl Zeiss Microscopy**, Carl-Zeiss-Straße 56, 73447 Oberkochen, Germany) and FEI Tecnai G2 T20 transmission electron microscopy (TEM) (**FEI Company**, 5350 NE Dawson Creek Drive, Hillsboro, Oregon 97124 USA). X-ray diffraction (XRD) measurement was used to characterize the crystallinity and crystal orientation of ZnO NRAs. PL spectroscopy was measured in a standard backscattering configuration where the light emission from top surface of the ZnO NRAs was collected, using a continuous-wave He-Cd laser (*λ *= 325 nm) as the excitation source.

## Results and discussion

**Figure 1:**
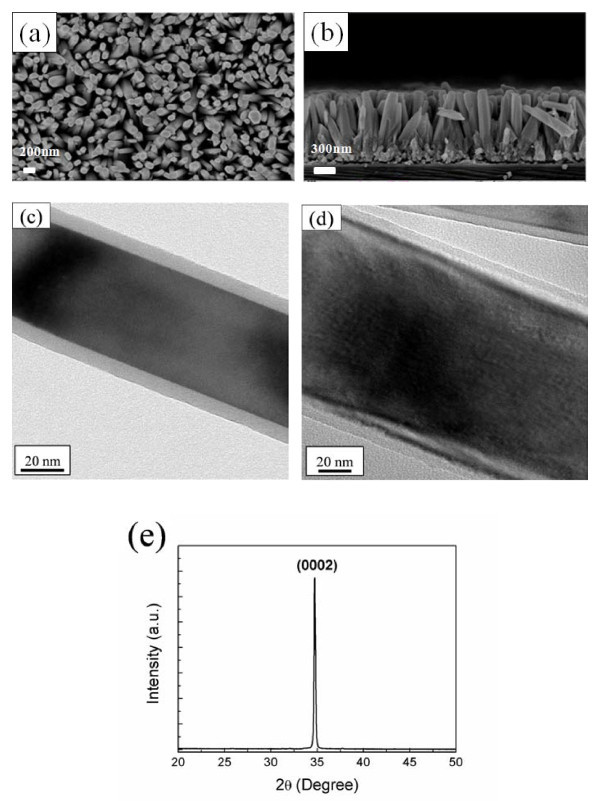
**SEM images, TEM images, and XRD pattern**. (**a**) Top-viewed and (**b**) cross-sectional SEM images of as-grown ZnO NRAs, (**c**) TEM image of the ZnO core with approximately 5 nm Al_2_O_3 _shell, (**d**) TEM image of the ZnO core with approximately 10 nm ZnO shell, and (**e**) XRD pattern of as-grown ZnO NRAs.

Top-viewed and cross-sectional SEM images of as-grown ZnO NRAs are shown in Figure 1a,b, respectively. The diameter of ZnO nanorods is in the range of 90 to 100 nm, and the length is about 1 μm. The substrate-bound NRAs were mechanically scraped, sonicated in ethanol, and deposited on carbon-coated copper grids for TEM characterization. Figure 1c,d shows low-magnification TEM images of ZnO/Al_2_O_3 _and ZnO/ZnO core-shell nanorods, indicating the uniformity in both of the core and shell layers. It can be seen that about 5 nm Al_2_O_3 _and 10 nm ZnO shell layers were deposited upon the surface of ZnO nanorods, demonstrating high conformality of the ALD technique. XRD pattern of as-grown ZnO NRAs is shown in Figure 1e, and the only dominant peak corresponding to (0002) plane was observed in the spectrum, revealing that ZnO nanorods are highly *c*-axis orientated. Moreover, it was noted that ZnO NRAs cannot be synthesized on (100) Si wafers without the ZnO seed layer.

**Figure 2:**
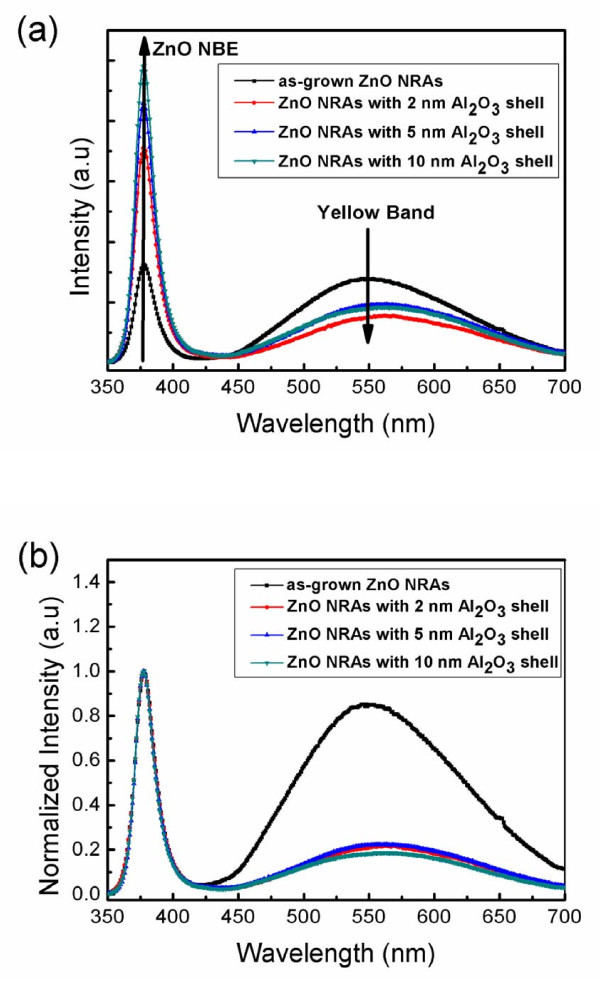
**PL spectra**. (**a**) Room-temperature PL spectra of as-grown ZnO NRAs and those coated with Al_2_O_3 _shell layers of different thicknesses. (**b**) Normalized PL spectra of (a). The PL spectra were normalized to the peak intensity of the NBE emission.

Figure 2a shows the room-temperature PL spectra of as-grown ZnO NRAs and those coated with the Al_2_O_3 _shell layers. Both the NBE emission (*λ *≈ 380 nm) and deep-level band associated with the oxygen interstitials (O_i_) (*λ *≈ 550 nm, yellow band) [22] were observed in the as-grown ZnO NRAs and ZnO/Al_2_O_3 _core-shell NRAs. As compared with as-grown ZnO NRAs, the NBE emission was significantly enhanced and the deep-level band was suppressed for the samples coated with Al_2_O_3 _shell layers. The intensity of NBE emission grows along with the increase of the Al_2_O_3 _shell-layer thickness. The deep-level band also increases slightly with the thickness of the Al_2_O_3 _shell layer. The PL spectra normalized to the peak intensity of each NBE emission are shown in Figure 2b. It can be seen that the ratio of the deep-level band to the NBE emission of the samples coated with Al_2_O_3 _shell layers is much smaller than that of as-grown ZnO NRAs. It may be also noted that the ratio of deep-level band to the NBE emission is almost identical for the ZnO/Al_2_O_3 _core-shell NRAs with different shell-layer thickness, suggesting that the same mechanism governs the increase of the NBE and deep-level emissions with the Al_2_O_3 _shell-layer thickness.

**Figure 3:**
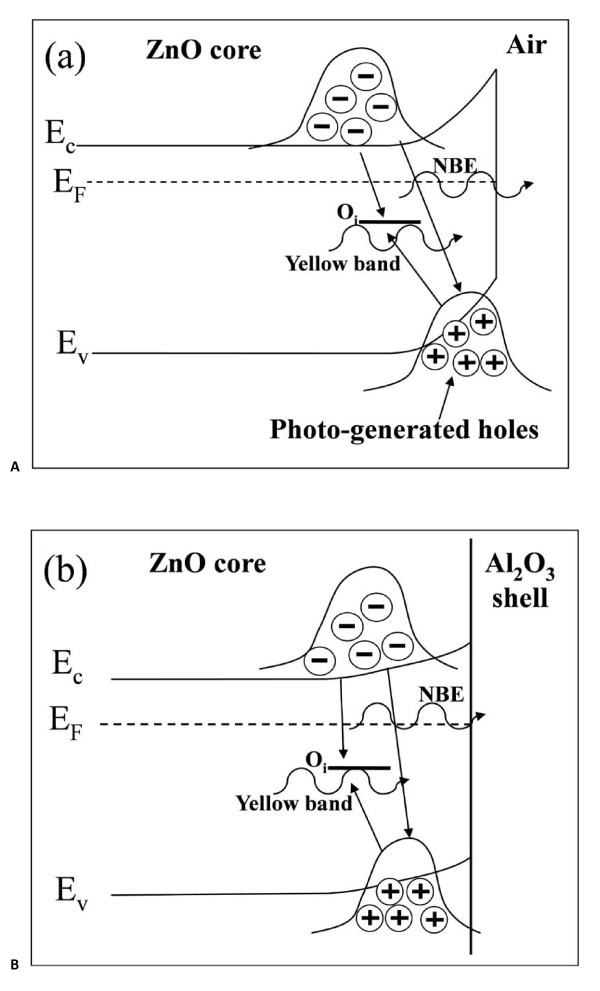
**Band diagrams**. Schematic band diagrams of (a) as-grown ZnO NRAs and (b) ZnO/Al_2_O_3 _core-shell NRAs.

As compared with the deep-level band of as-grown ZnO NRAs, the considerable suppression of the deep-level luminescence by the deposition of Al_2_O_3 _shell layers, as shown in Figure 2a,b, can be ascribed to the decrease in the density of oxygen interstitials at the surface of ZnO core [36]. The residual deep-level emission from the ZnO/Al_2_O_3 _core-shell NRAs may mainly originate from the oxygen interstitials inside the ZnO core. On the other hand, the remarkable enhancement of the ZnO NBE emission by depositing Al_2_O_3 _shell layers can be attributed to the flat-band effect [27,37]. Negatively charged oxygen ions may adsorb on the surface of as-grown ZnO nanorods, resulting in a depletion region near the surface [38]. Weber et al. have reported that the width of depletion region is about 20 nm [39], which is smaller than the diameter of the ZnO nanorods (approximately 100 nm) prepared in this study. This depletion region can be regarded as an upward band bending toward the surface as presented in the band diagram shown in Figure 3a. When the ZnO NRAs are irradiated by the pumping laser beam, the photo-generated holes are inclined to accumulate near the surface, and the photo-generated electrons tend to reside inside the core. As a result, the wavefunctions of electrons and holes are separated from each other, lowering the probability of radiative recombination to yield NBE emission. However, as plotted schematically in Figure 3b, the Al_2_O_3 _shell layer would eliminate the oxygen ions adsorbed on the ZnO surface and hence reduce the band bending near the interface [27]. Therefore, the overlap between the wavefunctions of electrons and holes in the ZnO core is increased, leading to the enhancement of NBE emission. The increase of the Al_2_O_3 _shell-layer thickness from 2 to 10 nm may further lower the band bending near the interface and thus enhance the wavefunction overlap, resulting in the increase in NBE emission with the thickness of the Al_2_O_3 _shell layer. The same argument also holds for the carrier recombination through the deep-level states inside the ZnO core. As illustrated in Figure 3a,b, the flat-band effect may also enhance the deep-level emission around *λ *≈ 550 nm originating from the oxygen interstitials inside the ZnO core due to the increase of the wavefunction overlap. Accordingly, as shown in Figure 2b, the normalized PL spectra present almost the same ratio of the deep-level band to the NBE emission for the NRAs with different Al_2_O_3 _shell-layer thickness, indicating that the increase of the Al_2_O_3 _shell-layer thickness enhances both the NBE and deep-level emissions due to the flat-band effect.

**Figure 4:**
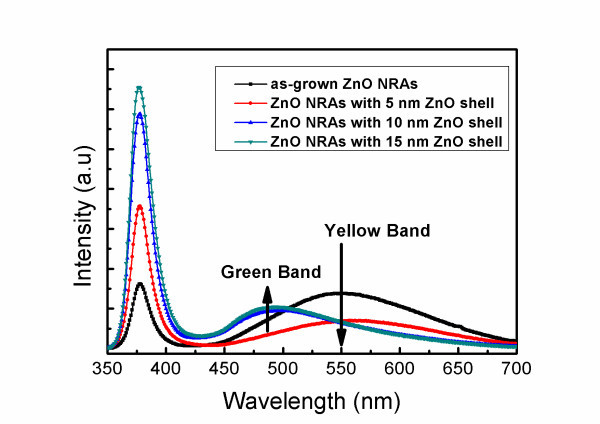
**PL spectra**. Room-temperature PL spectra of as-grown ZnO NRAs and those coated with ZnO shell layers of different thicknesses.

To further investigate the effect of surface band bending in ZnO nanorods, we conducted the PL measurement on ZnO/ZnO core-shell NRAs with different thicknesses of ZnO shell layers. Since the absorption coefficient of ZnO at *λ *= 325 nm is about 1.5 × 10^5 ^cm^-1 ^[40] and the estimated penetration depth is approximately 67 nm, both ZnO cores and ZnO shells could be excited by the He-Cd laser during the PL measurement. Figure 4 shows the PL spectra of the as-grown ZnO NRAs and ZnO/ZnO core-shell NRAs at room temperature. As compared with as-grown ZnO NRAs, the NBE emission was enhanced and the deep-level band around 550 nm was suppressed after a 5-nm-thick ZnO shell layer was deposited. This can be realized that the ZnO shell layer could give rise to the increase of the flat-band region in the ZnO core and the reduction in the density of oxygen interstitials at the surface of ZnO core. Similar to the ZnO/Al_2_O_3 _core-shell NRAs, the residual deep-level band around *λ *≈ 550 nm of the NRAs coated with a 5-nm-thick ZnO shell layer can be attributed to light emission from the oxygen interstitials inside the ZnO core.

**Figure 5:**
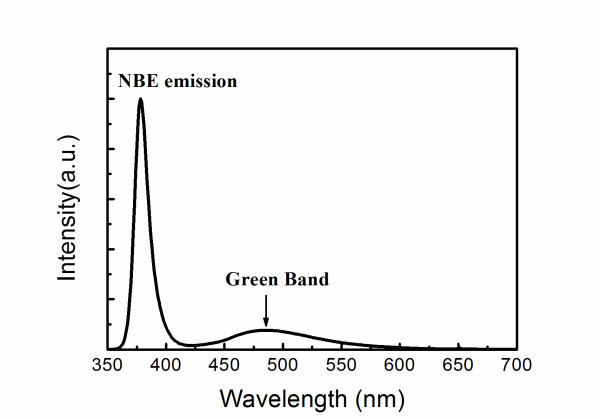
**PL spectrum**. Room-temperature PL spectrum of the ZnO seed layer grown by ALD.

**Figure 6:**
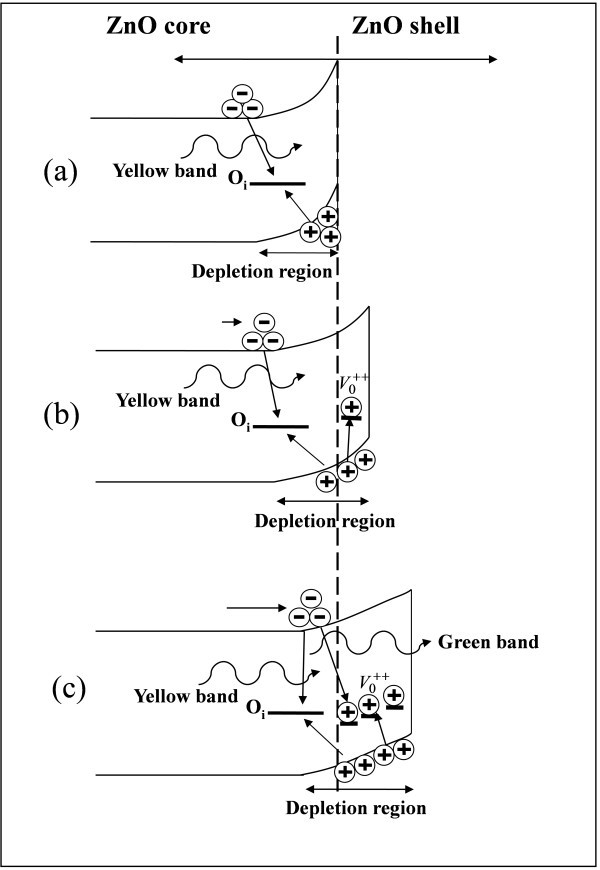
**Band diagrams**. Schematic band diagram of ZnO/ZnO core-shell structures with ZnO shell layers of different thicknesses.

Figure 4 also presents the remarkable shift of the defect-related luminescence, from the yellow band (approximately 550 nm) to the green band (approximately 490 nm), as the thickness of the ZnO shell layer is greater than 10 nm. This green band can be also found in the PL spectrum of the ZnO seed layer grown by ALD, as shown in Figure 5, suggesting that the green band may originate from the ALD ZnO shell layer. It has been reported that the green band arises from the recombination of the electrons in the conduction band and the holes trapped by the $V_{0}^{+}$ center (one electron at the site of oxygen vacancy) [27,41]. As shown schematically in Figure 6a, the photo-generated holes are accumulated near the surface of ZnO nanorods due to the surface band bending. As a 5-nm-thick ZnO shell layer was deposited by ALD, the $V_{0}^{+}$ centers in the ZnO shell layer trap the photo-generated holes and then convert to $V_{0}^{++}$, as illustrated in Figure 6b. However, the band bending depletes the electrons near the surface so as to suppress the recombination of the electrons and the $V_{0}^{++}$ centers. As a result, the green band associated with $V_{0}^{++}$ did not appear; instead, the yellow band from the oxygen interstitials inside the ZnO core was observed in the PL spectrum. Figure 6c shows that the extension of flat-band region in the ZnO core can reach the ZnO/ZnO core-shell interface as the ZnO shell layer is thick enough. Therefore, the $V_{0}^{++}$ centers can recombine with the electrons in the conduction band to yield the green luminescence. As a result, the green band dominates over the yellow band as the ZnO shell-layer thickness is greater than 10 nm, as shown in the PL spectra in Figure 4.

## Conclusion

In summary, the ZnO/Al_2_O_3 _and ZnO/ZnO core-shell NRAs have been prepared using the aqueous chemical synthesis and the conformal ALD technique. The deep-level emission around *λ *≈ 550 nm from the oxygen interstitials at the surface of ZnO cores was suppressed by the Al_2_O_3 _and ZnO shell layers. The shell layers also reduce the surface band bending, leading to the increase in overlap of the wavefunctions of electrons and holes in the ZnO core. Therefore, the NBE emission at *λ *≈ 380 nm and the deep-level band around *λ *≈ 550 nm from the oxygen interstitials inside the core were enhanced by the shell layers. Furthermore, the shift of defect-related emissions from the ZnO/ZnO core-shell NRAs was observed due to the competition between light emissions from the oxygen interstitials inside the ZnO core and the oxygen vacancies in the ZnO shell. As the thickness of the ZnO shell layer increased, the green luminescence (*λ *≈ 490 nm) originating from the oxygen vacancies in the shell dominated over the yellow band (*λ *≈ 550 nm) associated with the oxygen interstitials inside the ZnO core due to the flat-band effect. The results indicate that the shell layers prepared by ALD have significant influence both on the NBE and defect-related emissions of the ZnO NRAs.

## Competing interests

The authors declare that they have no competing interests.

## Authors' contributions

All the authors contributed to the writing of the manuscript. WCS and YCY carried out the experiments under the instruction of MJC. CTK performed the TEM measurement. All authors read and approved the final manuscript.
